# Comprehensive behavioral analysis of mice deficient in Rapgef2 and Rapgef6, a subfamily of guanine nucleotide exchange factors for Rap small GTPases possessing the Ras/Rap-associating domain

**DOI:** 10.1186/s13041-018-0370-y

**Published:** 2018-05-10

**Authors:** Kazuhiro Maeta, Satoko Hattori, Junji Ikutomo, Hironori Edamatsu, Shymaa E. Bilasy, Tsuyoshi Miyakawa, Tohru Kataoka

**Affiliations:** 1 Division of Molecular Biology, Department of Biochemistry and Molecular Biology, Kobe University Graduate School of Medicine, 7-5-1 Kusunoki-cho, Chuo-ku, Kobe, 650-0017 Japan; 2 Division of Systems Medical Science, Institute for Comprehensive Medical Science, Fujita Health University, 1-98 Dengakugakubo, Kutsukake-cho, Toyoake, Aichi 470-1192 Japan; 3 Present address: Department of Neurotherapeutics, Osaka University Graduate School of Medicine, 2-2Yamadaoka, Suita, Osaka, 565-0871 Japan; 4 Present address: Department of Biochemistry, Faculty of Pharmacy, Suez Canal University, El-shikh Zayed, Ismailia, 41522 Egypt

**Keywords:** Rap small GTPases, Guanine nucleotide exchange factors, Rapgef2, Rapgef6, Behavioral analysis, Schizophrenia

## Abstract

Rapgef2 and Rapgef6 define a subfamily of guanine nucleotide exchange factors for Rap small GTPases, characterized by the possession of the Ras/Rap-associating domain. Previous genomic analyses suggested their possible involvement in the etiology of schizophrenia. We recently demonstrated the development of an ectopic cortical mass (ECM), which resembles the human subcortical band heterotopia, in the dorsal telencephalon-specific *Rapgef2* conditional knockout (*Rapgef2*-cKO) brains. Additional knockout of *Rapgef6* in *Rapgef2*-cKO mice resulted in gross enlargement of the ECM whereas knockout of *Rapgef6* alone (*Rapgef6*-KO) had no discernible effect on the brain morphology. Here, we performed a battery of behavioral tests to examine the effects of *Rapgef2* or *Rapgef6* deficiency on higher brain functions. *Rapgef2*-cKO mice exhibited hyperlocomotion phenotypes. They showed decreased anxiety-like behavior in the elevated plus maze and the open-field tests as well as increased depression-like behavior in the Porsolt forced swim and tail suspension tests. They also exhibited increased sociability especially in novel environments. They showed defects in cognitive function as evidenced by reduced learning ability in the Barnes circular maze test and by impaired working memory in the T maze tests. In contrast, although Rapgef6 and Rapgef2 share similarities in biochemical roles, *Rapgef6*-KO mice exhibited mild behavioral abnormalities detected with a number of behavioral tests, such as hyperlocomotion phenotype in the open-field test and the social interaction test with a novel environment and working-memory defects in the T-maze test. In conclusion, although there were differences in their brain morphology and the magnitude of the behavioral abnormalities, *Rapgef2*-cKO mice and *Rapgef6*-KO mice exhibited hyperlocomotion phenotype and working-memory defect, both of which could be recognized as schizophrenia-like behavior.

## Introduction

Rap proteins (Rap1A, Rap1B, Rap2A, Rap2B and Rap2C) belong to the Ras-family of small GTPases and are implicated in the regulation of a variety of cellular phenomena including proliferation, adhesion, polarity and endocytosis [1]. In the nervous system, Rap proteins have been demonstrated to play important roles in neural development during embryogenesis as well as in synaptic remodeling and plasticity in differentiated neurons [2–8]. During embryogenesis, Rap1 function as master regulators of neural cell polarity in the neocortical development and their loss leads to severe brain malformations in mice [5]. In differentiated neurons, Rap1 and Rap2 are involved in long-term depression, cortico-amygdala plasticity, fear learning, spatial learning and fear extinction [6, 9–12]. Guanine nucleotide exchange factors (GEFs) function as activators of small GTPases by catalyzing the release of GDP from the inactive GDP-bound forms, thereby accelerating GTP loading to yield the active GTP-bound forms [13]. To date, more than 10 GEFs specific for Rap have been reported. They are regulated by distinct mechanisms and responsible for differential regulation of Rap1 activity in spatial, temporal and cell-type-specific manners [1]. A number of Rap1-GEFs are known to play important roles in development and function in the nervous system. For example, Rapgef4, also named Epac2 and cAMP-GEF1, activated by cAMP binding promotes spine shrinkage, resulting in synapse structural destabilization in cultured rat cortical neurons [14]. Moreover, a mutation in the *Rapgef4* gene was associated with autism in humans, and the overexpression of this Rapgef4 mutant resulted in an impairment of basal dendrite maintenance in mice [15]. Combined deletion of Rapgef4 and its paralogue Rapgef3, also named Epac1 and cAMP-GEF1, in the forebrain caused defects in spatial learning and social interaction in mice [16]. Rapgef1, also named C3G, is responsible for Rap1 activation downstream of the Reelin signaling and plays a crucial role in neural development, in particular neuronal migration [17].

Rapgef2, also named RA-GEF-1, PDZ-GEF1, CNrasGEF and nRapGEP [18–21], and Rapgef6, also named RA-GEF-2 and PDZ-GEF2 [22], constitute a unique Rap1-GEF subfamily characterized by the possession of the Ras/Rap-associating (RA) domains capable of associating with the GTP-bound forms of Rap1 and M-Ras and the PSD-95/DlgA/ZO-1 (PDZ) domains. We previously showed that dorsal telencephalon-specific conditional *Rapgef2* knockout (*Rapgef2*-cKO) mice developed severe brain malformations including an ectopic cortical mass (ECM) extending throughout the rostro-caudal axis of the cerebral hemisphere, enlargement of the lateral ventricles, interruption of the pyramidal cells in the hippocampal CA1 region and agenesis of interhemispheric connections [23–25]. We also reported that *Rapgef2*-cKO mice exhibited a lower seizure threshold to pilocarpine-induced status epilepticus [24]. Further analyses of their brain development revealed that they exhibit severe defects in formation of apical surface adherence junctions and in the development of radial glial cells (RGCs) [26]. Additional knockout of *Rapgef6* in *Rapgef2*-cKO mice (*Rapgef2/6*-dKO) resulted in marked enlargement of the ECM and aggravation of the RGC developmental defects, suggesting that Rapgef6 shares neural functions with Rapgef2 [26]. On the other hand, knockout of *Rapgef6* alone (*Rapgef6-*KO) had no discernible effect on the brain morphology [26, 27]. Moreover, human genetic studies in a cohort of schizophrenia patients showed strong genetic association of rare inherited copy number variations involving *RAPGEF2* and *RAPGEF6* with schizophrenia [28, 29]. Moreover, the ECM formed in *Rapgef2*-cKO mice resembles that found in human subcortical band heterotopia patients, who display symptoms of mental retardation and epilepsy [30]. In *Rapgef2*-cKO mice, the malformation of the commissural system and agenesis of the corpus callosum (CC) [24], were also reported to be associated with schizophrenia-like behavior in mice carrying mutations in the *Disrupted in Schizophrenia-1* (*DISC-1*) gene [31–34]. These results prompted us to perform comprehensive behavioral analysis in order to gain insights into the role of these Rap activators in the regulation of higher brain functions.

## Results

### General health and general behavioral characteristics

**Fig. 1:**
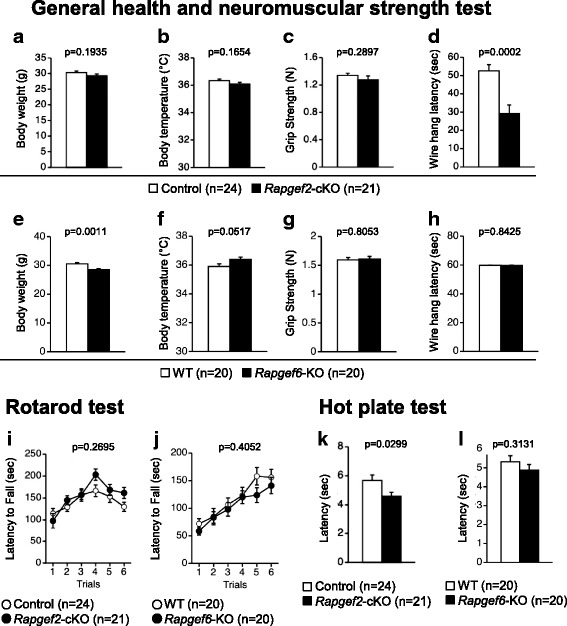
Physical characteristics and neurological screening. **a-h** General health and neuromuscular strength tests. Bodyweight (**a**, **e**), body temperature (**b**, **f**), neuromuscular strength determined by the grip strength test (**c**, **g**), and that determined by the wire hang test (**d**, **h**) are shown. **i**, **j** Rotarod test. **k**, **l** Hot plate test. *n* indicates the number of individuals tested. *p* values were determined as described in *Methods*

*Rapgef2-*cKO mice, *Rapgef2* was specifically disrupted in the dorsal telencephalon, were created by mating *Rapgef2*^flox/flox^ mice with *Emx1*^cre/+^ mice expressing Cre recombinase under the control of the *Emx1* promoter [24]. To assess the effects of *Rapgef2* deficiency, we compared *Rapgef2-*cKO mice and *Rapgef2*^flox/flox^;*Emx1*^+/+^ mice in this study. We also assessed the effects of *Rapgef6* deficiency by comparing *Rapgef6*-KOmice and *Rapgef6*^+/+^ (wild-type) mice [35]. We performed the experiments using *Rapgef2-*cKO mice and *Rapgef2*^flox/flox^;*Emx1*^+/+^ mice (herein after referred to as control mice), and then performed those using *Rapgef6*-KO mice and wild-type mice. In our experimental model, it was difficult to compare between control mice and wild-type mice because of the differences in the factors affecting the results, such as the number of backcrossing and environmental factors. In *Rapgef2-*cKO mice, the body weight and temperature were comparable to those in the control mice (Fig. 1a, F_1,43_ = 1.745, *p* = 0.1935; Fig. 1b, F_1,43_ = 1.991, *p* = 0.1654). Although the grip strength was not significantly affected by *Rapgef2* genotype (Fig. 1c, F_1,43_ = 1.149, *p* = 0.2897), the wire-hang test demonstrated a significant decrease in the latency to fall in *Rapgef2*-cKO mice (Fig. 1d, F_1,43_ = 16.785, *p* = 0.0002). In *Rapgef6*-KO mice, the bodyweight was significantly lighter (Fig. 1e, F_1,38_ = 12.541, *p* = 0.0011) and the body temperature was relatively higher than the wild-type littermates (Fig. 1f, F_1,38_ = 4.036, *p* = 0.0517). *Rapgef6* genotype did not seem to affect the grip strength and the latency to fall in the wire-hang test (Fig. 1g, F_1,38_ = 0.062, *p* = 0.8053; Fig. 1h, F_1,38_ = 0.04, *p* = 0.8425, respectively). In the accelerating rotarod test, although the differences associated with the *Rapgef2* or *Rapgef6* genotype were not statistically significant, we observed a slight tendency for increased latency to fall in *Rapgef2*-cKO mice (Fig. 1i, genotype effect, F_1,43_ = 1.251, *p* = 0.2695; trial effect, F_5,215_ = 14.382, *p* < 0.0001; genotype × trial interaction, F_1,43_ = 2.051, *p* = 0.0728; 4th trial, genotype effect, F_1,43_ = 3.86, *p* = 0.0559; 5th trial, genotype effect, F_1,43_ = 0.649, *p* = 0.4249; 6th trial, genotype effect, F_1,43_ = 3.852, *p* = 0.0562; Fig. 1j, genotype effect, F_1,38_ = 0.708, *p* = 0.4052; trial effect, F_5,190_ = 34.155, *p* < 0.0001; genotype × trial interaction, F_5,190_ = 1.127, *p* = 0.3477). In the hot plate pain test, the latency to the first hind-paw response was significantly decreased in *Rapgef2*-cKO mice (Fig. 1k, F_1,43_ = 5.043, *p* = 0.0299) but not in *Rapgef6-*KO mice (Fig. 1l, F_1,38_ = 1.045, *p* = 0.3131).

### Effects on locomotor activity and anxiety-like behavior

**Fig. 2:**
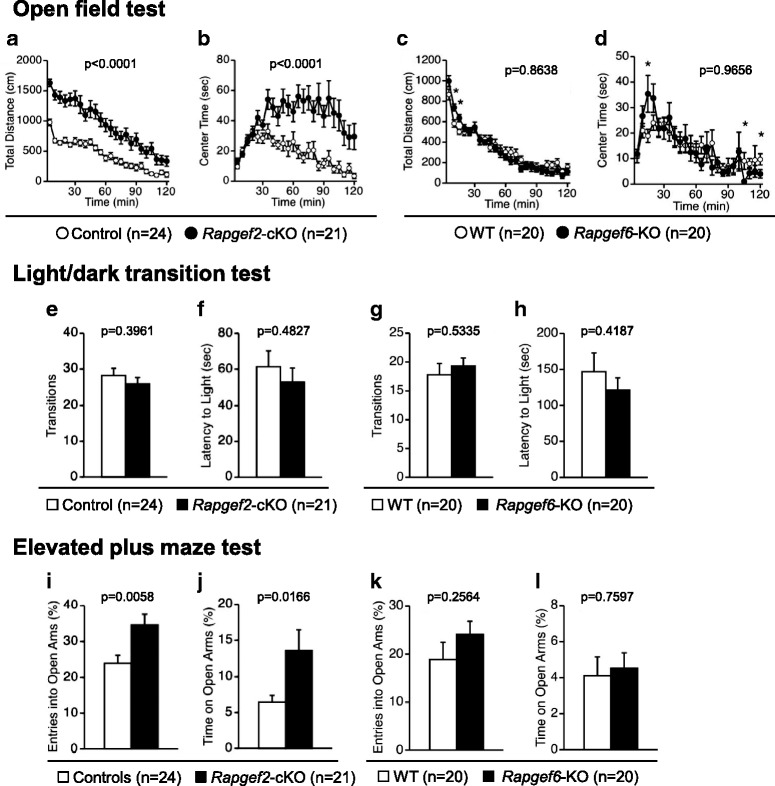
Assessment for locomotor activity and anxiety-like behavior. **a**-**d** Open-field test in a novel environment. Total distance traveled (**a**, **c**) and time spent in arena center (**b**, **d**) were determined. **e**-**h** Light/dark transition test. The number of light/dark transitions (**e**, **g**) and the first latency to enter the light chamber (**f**, **h**) were determined. **i-l** Elevated plus maze test. Percentages of entries into the open arms (**i**, **k**) and those of time spent in the open arms (**j**, **l**) were determined. *n* indicates the number of individuals tested. *p* values were determined as described in *Methods*. *, *p* < 0.05

We performed the open field test with a novel environment to examine the effects on the locomotor activity. In all of the time windows examined, *Rapgef2-*cKO mice traveled longer in distance than control mice (Fig. 2a, genotype effect, F_1,43_ = 36.348, *p* < 0.0001; time effect; F_23,989_ = 70.365, *p* < 0.0001; genotype ×time interaction, F_23,989_ = 5.174, *p* < 0.0001). In contrast, *Rapgef6*-KO mice did not clearly exhibit difference in the total distance traveled (Fig. 2c, genotype effect, F_1,38_ = 0.03, *p* = 0.8638). However, the time-dependent changes appeared to be affected by the *Rapgef6* genotype (Fig. 2c, time effect, F_23,874_ = 84.27, *p* < 0.0001; genotype × time interaction, F_23,874_ = 2.350, *p* = 0.0004). These results suggest that *Rapgef2-*cKO mice strongly exhibited hyperlocomotion phenotypes whereas *Rapgef6*-KO mice weakly exhibited hyperlocomotion phenotypes consistent with previous observations [27].

In the open field test, the time spent exploring the center area is generally considered as an index of anxiety. The time spent in the arena center was significantly increased in *Rapgef2*-cKO mice (Fig. 2b, F_1,43_ = 19.658, *p* < 0.0001). The time spent in the arena center was also affected by the *Rapgef6* genotype in a time-dependent manner (Fig. 2d, genotype effect, F_1,38_ = 0.002, *p* = 0.9656; genotype × time interaction, F_23,874_ = 1.618, *p* = 0.0334; time effect, F_23,874_ = 10.426, *p* < 0.0001). *Rapgef6*-KO mice spent more time in the arena center in the initial phase of the test although they stayed in the peripheral arena in later phases (Fig. 2d).

To further assess the impacts on anxiety-like behavior, we performed a light/dark transition and elevated plus maze tests. The light/dark transition test failed to detect the changes associated with the genotypes of *Rapgef2* or *Rapgef6*; the number of transitions between the light and dark chambers was not significantly different among the examined groups (*Rapgef2*-cKO, Fig. 2e, F_1,43_ = 0.735, *p* = 0.3961; *Rapgef6*-KO, Fig. 2g, F_1,38_ = 0.395, *p* = 0.5335), the latency to first enter the light chamber (*Rapgef2*-cKO, Fig. 2f, F_1,43_ = 0.501, *p* = 0.4827; *Rapgef6*-KO, Fig. 2h, F_1,38_ = 0.669, *p* = 0.4187).

In an elevated plus maze test, *Rapgef2*-cKO mice preferred the open arms as judged by increased percentage of entries into the open arms (Fig. 2i, F_1,42_ = 8.453, *p* = 0.0058) and in the percentage of spent time in the open arms (Fig. 2j, F_1,42_ = 6.229, *p* = 0.0166). On the other hand, *Rapgef6*-KO mice did not show any significant difference compared to wild-type mice in the open arms entry percentage and the percentage of spent time in the open arms (Fig. 2k, F_1,38_ = 1.328, *p* = 0.2564; Fig. 2l, F_1,38_ = 0.095, *p* = 0.7597, respectively).

### Effects on depression-like behavior

**Fig. 3:**
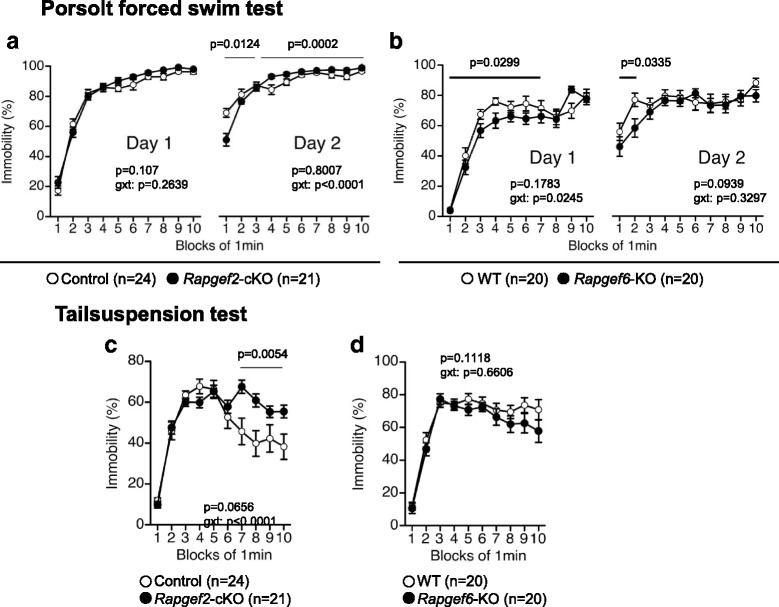
Assessment of depression-like behavior. **a**, **b** Porsolt forced swim test. Percentage in immobility at days 1 and 2 and *p* values for genotype × time interaction (*g* × *t*) are shown. **c**, **d** Tail suspension test. Percentages of immobility were determined. *p* value for genotype × time interaction (*g* × *t*) are shown

To assess depression-like behavior, we employed the Porsolt forced swim test. We used the protocol in which the tests are performed in consecutive 2 days. This protocol, originally developed for the experiments using rats, is suitable for interpreting behavioral phenotypes more comprehensively. On day 1, *Rapgef2*-cKO mice did not show no significant genotype effect in the immobility (Fig. 3a, *left*, genotype effect, F_1,43_ = 2.71, *p* = 0.107; genotype × time interaction, F_9,387_ = 1.248, *p* = 0.2639). On day 2, *Rapgef2*-cKO mice exhibited increased immobility during the 4-10 min time windows with an inverse trend seen during the 1-3 min time windows (Fig. 3a, *right*, genotype effect, F_1,43_ = 0.065, *p* = 0.8007; genotype × time interaction, F_9,387_ = 7.024, *p* < 0.0001; genotype effect, 1-3 min time windows, F_1,43_ = 6.82, *p* = 0.0124; 4-10 min time windows, F_1,43_ = 17.252, *p* = 0.0002). In contrast, *Rapgef6*-KO mice exhibited decreased immobility during the first 1-7 min at day 1 (Fig. 3b, *left*, genotype effect, F_1,38_ = 1.881, *p* = 0.1783; genotype × time interaction, F_9,342_ = 2.158, *p* = 0.0245; genotype effect, 1-7 min time windows, F_1,38_ = 5.089, *p* = 0.0299). At day 2, *Rapgef6*-KO mice exhibited decreased immobility specifically in the initial phases of the test (Fig. 3b, *right*, genotype effect, F_1,38_ = 2.952, *p* = 0.0939; genotype × time interaction, F_9,342_ = 1.146, *p* = 0.3297; genotype effect, 1-2 min time windows, F_1,38_ = 4.865, *p* = 0.0335).

To further assess depression-like phenotype, we performed the tail suspension test. *Rapgef2*-cKO mice exhibited a significant increase in the percentage of immobility, during the 7-10 min time frame (Fig. 3c, genotype effect, F_1,43_ = 3.569, *p* = 0.0656; genotype × time interaction, F_9,387_ = 4.481, *p* < 0.0001; 7-10 min time windows, F_1,43_ = 8.574, *p* = 0.0054). In contrast, *Rapgef6*-KO mice did not exhibit any significant differences compared to wild-type mice (Fig. 3d, genotype effect, F_1,38_ = 2.651, *p* = 0.1118; genotype × time interaction, F_9,342_ = 0.752, *p* = 0.6606).

### Effects on startle response and prepulse inhibition test

**Fig. 4:**
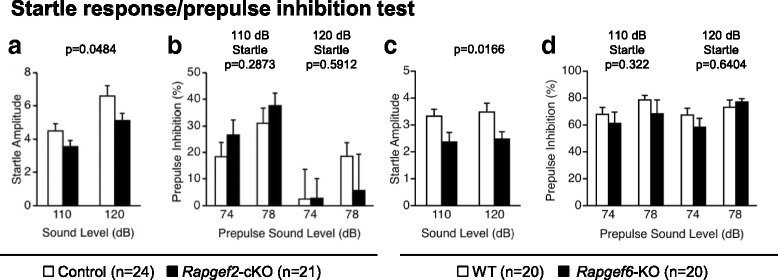
Startle response/prepulse inhibition test. Amplitudes of startle response (**a**, **c**) and percentages of prepulse inhibition (**b**, **d**) were determined

Alterations in *RAPGEF2* and *RAPGEF6* were reported in a group of schizophrenic patients [28, 29]. Therefore, we performed the prepulse inhibition test to investigate whether *Rapgef2*-cKO mice and *Rapgef6*-KO mice exhibited schizophrenia-like phenotype. *Rapgef2*-cKO mice and *Rapgef6*-KO mice significantly exhibited a decreased startle amplitude when tested with the auditory stimuli at the sound pressure levels of 110 and 120 dB (Fig. 4a, F_1,43_ = 4.129, *p* = 0.0484; Fig. 4c, F_1,38_ = 6.287, *p* = 0.0166, respectively). However, we failed to detect any significant effects of *Rapgef2* or *Rapgef6* deficiency in the prepulse inhibition test (Fig. 4b, 110 dB startle, F_1,43_ = 1.161, *p* = 0.2873; 120 dB startle, F_1,43_ = 0.293, *p* = 0.5912; Fig. 4d, 110 dB startle, F_1,38_ = 1.007, *p* = 0.322; 120 dB startle, F_1,38_ = 0.222, *p* = 0.6404, respectively).

### Effects on social behavior

**Fig. 5:**
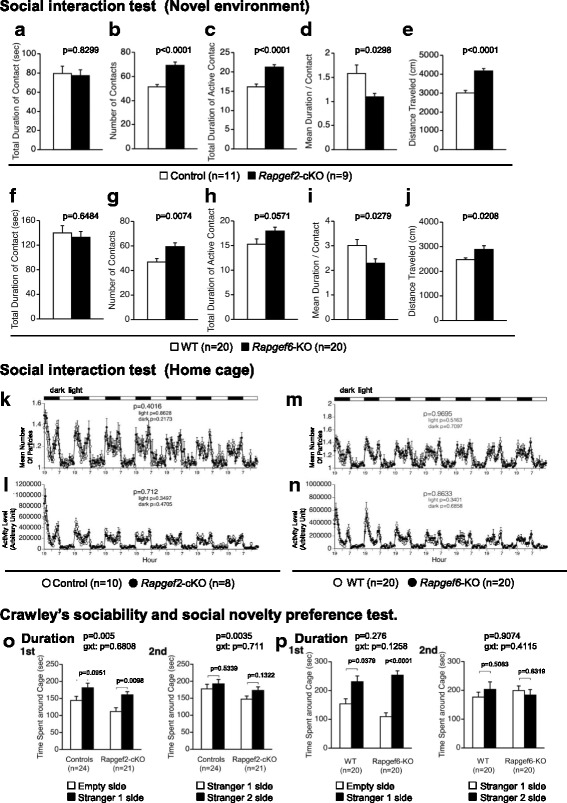
Assessment for social behavior. **a**-**j** Social interaction test in a novel environment. Total duration of contacts (**a**, **f**), the number of contacts (**b**, **g**), total duration of active contacts (**c**, **h**), mean duration of each contact (**d**, **i**), and total distance traveled (**e**, **j**) were determined. **k**-**n** Social interaction test in a home cage. Means of the numbers of the particles formed with the mice tested were determined (**k**, **m**). The activity levels were calculated (**l**, **n**). **o**, **p** Crawley’s sociability and social novelty preference test. Time spent around the indicated cages was determined. In *1st*, a wire cage keeping a stranger mouse (*Stranger 1 side*) and an empty wire cage (*Empty side*) were used. In *2nd*, a cage keeping a familiar mouse (*Stranger 1 side*) and a cage keeping another stranger mouse (*Stranger 2 side*) were used. *p* values for genotype effects are shown on the top of each bargraph

To assess the effects of *Rapgef2* and *Rapgef6* genotypes on social behavior, a social interaction test with novel environment was performed (Fig. 5a-j). Although the *Rapgef2* genotype did not affect the total duration of contacts (Fig. 5a, F_1,18_ = 0.048, *p* = 0.8299), *Rapgef2*-cKO mice exhibited significant increases in the number of contacts (Fig. 5b, F_1,18_ = 28.989, *p* < 0.0001) and in the total duration of active contacts (Fig. 5c, F_1,18_ = 27.519, *p* < 0.0001) accompanied by a decrease in the duration of each contact (Fig. 5d, F_1,18_ = 5.566, *p* = 0.0298). Further, the distance traveled was increased in *Rapgef2*-cKO mice (Fig. 5e, F_1,18_ = 39.017, *p* < 0.0001). *Rapgef6*-KO mice exhibited behavior trends similar to those of *Rapgef2*-cKO mice. Even though the total duration of contacts was not affected (Fig. 5f, F_1,18_ = 0.215, *p* = 0.6484), *Rapgef6*-KO mice showed increases in the number of contacts (Fig. 5g, F_1,18_ = 9.088, *p* = 0.0074) and in the total duration of active contacts (Fig. 5h, F_1,18_ = 4.132, *p* = 0.0571) accompanied by a decrease in the duration of each contact (Fig. 5i, F_1,18_ = 5.719, *p* = 0.0279). Additionally, the distance traveled was increased in *Rapgef6*-KO mice (Fig. 5j, F_1,18_ = 6.421, *p* = 0.0208).

Next, a social interaction test using a home cage was performed (Fig. 5k-n). We did not observe any statistically significant alteration depending on the genotype of *Rapgef2* or *Rapgef6* in the mean number of particles formed with mice examined in each time window (Fig. 5k, throughout the experimental period, F_1,16_ = 0.743, *p* = 0.4016; light period, F_1,16_ = 0.031, *p* = 0.8628; dark period, F_1,16_ = 1.65, *p* = 0.2173; Fig. 5m, throughout the experimental period, F_1,18_ = 0.002, *p* = 0.9695; light period, F_1,18_ = 0.438, *p* = 0.5163; dark period, F_1,18_ = 0.143, *p* = 0.7097) or in the activity levels (Fig. 5l, throughout the experimental period, F_1,16_ = 0.141, *p* = 0.712; light period, F_1,16_ = 0.928, *p* = 0.3497; dark period, F_1,16_ = 0.546, *p* = 0.4705; Fig. 5n, throughout the experimental period, F_1,18_ = 0.031, *p* = 0.8633; light period, F_1,18_ = 0.96, *p* = 0.3401; dark period, F_1,18_ = 0.169, *p* = 0.6858). These results seemed somewhat contradictory because hyperlocomotion phenotype in this test was not obvious in *Rapgef2*-cKO mice, which at the age of 18-24 weeks showed a marked increase in the locomotor activity in the open field test (Fig. 2a). Therefore, we performed the open field test using *Rapgef2*-cKO mice after the completion of the social interaction test, and found hyperlocomotion phenotype in aged (72-78 weeks old) *Rapgef2*-cKO mice (data not shown).

To further assess the social behavior, we performed Crawley’s sociability and social novelty preference tests (Fig. 5o, p), a well-designed method to investigate the effects of complex genetics on sociability and preference for social novelty [36, 37]. First, we performed the sociability test using the test equipment containing an empty wire cage and a wire cage apparatus keeping a stranger mouse (Stranger 1). *Rapgef2*-cKO mice spent a longer time around the cage containing Stranger 1 than around the empty cage, but such trends were relatively weak in control mice (Fig. 5o, *1st*, control mice, *t* = 1.741, df = 23, *p* = 0.0951; *Rapgef2*-cKO mice, *t* = 2.856, df = 20, *p* = 0.0098, Stranger 1 side vs. empty side, paired *t*-test). A two-way repeated measures ANOVA demonstrated that *Rapgef2*-cKO mice spent significantly shorter time around the cages (Fig. 5o, *1st*, genotype effect, F_1,43_ = 8.754, *p* = 0.005; genotype × cage interaction, F_1,43_ = 0.172, *p* = 0.6808). We next performed the social novelty preference test using the test equipment containing a wire cage keeping another unfamiliar mouse (Stranger 2) and a wire cage keeping Stranger 1 that had already been used in the preceding trials and thus became familiar to the mice subjected to this test (Fig. 5o, *2nd*). The time spent around the cage keeping Stranger 2 was not considerably different from the time spent around the cage keeping Stranger 1 regardless of the *Rapgef2* genotype (Fig. 5o, *2nd*, control mice, *t* = 0.622, df = 23, *p* = 0.5399; *Rapgef2*-cKO mice, *t* = 1.569, df = 20, *p* = 0.1322, Stranger 2 side vs. Stranger 1side, paired *t*-test). A two-way repeated measures ANOVA again demonstrated that *Rapgef2*-cKO mice spent significantly shorter time around the cages (Fig. 5o, *2nd*, genotype effect, F_1,43_ = 9.539, *p* = 0.0035; genotype × cage interaction, F_1,43_ = 0.139, *p* = 0.711).

We performed similar experiments to assess the effects of the *Rapgef6* genotype. *Rapgef6*-KO mice spent longer time around the cage containing Stranger 1 than around the empty cage, and similar trends were detected in wild-type control mice (Fig. 5p, *1st*, wild-type mice, *t* = − 2.231, df = 19, *p* = 0.0379; *Rapgef6*-KO mice, *t* = − 5.607, df = 19, *p* < 0.0001, Stranger 1 side vs. empty side, paired *t*-test). A two-way repeated measures ANOVA demonstrated that no significant genotype effect was observed in the time spent around cage between wild-type mice and *Rapgef6*-KO mice (Fig. 5p, *1st*, genotype effect, F_1,38_ = 1.221, *p* = 0.276; genotype × cage interaction, F_1,38_ = 2.451, *p* = 0.1258). In the social novelty preference test, the time spent around the cage keeping Stranger 2 was not significantly different from the time spent around the cage keeping Stranger 1 regardless of *Rapgef6* genotype (Fig. 5p, *2nd*, wild-type mice, *t* = − 0.674, df = 19, *p* = 0.5083; *Rapgef6*-KO mice, *t* = 0.487, df = 19, *p* = 0.6319, Stranger 2 side vs. Stranger 1 side, paired *t*-test). A two-way repeated measures ANOVA again demonstrated that no significant genotype effect was observed in the time spent around cage between wild-type mice and *Rapgef6*-KO mice (Fig. 5p, *2nd*, genotype effect, F_1,38_ = 0.014, *p* = 0.9074; genotype × cage interaction, F_1,38_ = 0.689, *p* = 0.4115).

### Effects on cognitive function

**Fig. 6:**
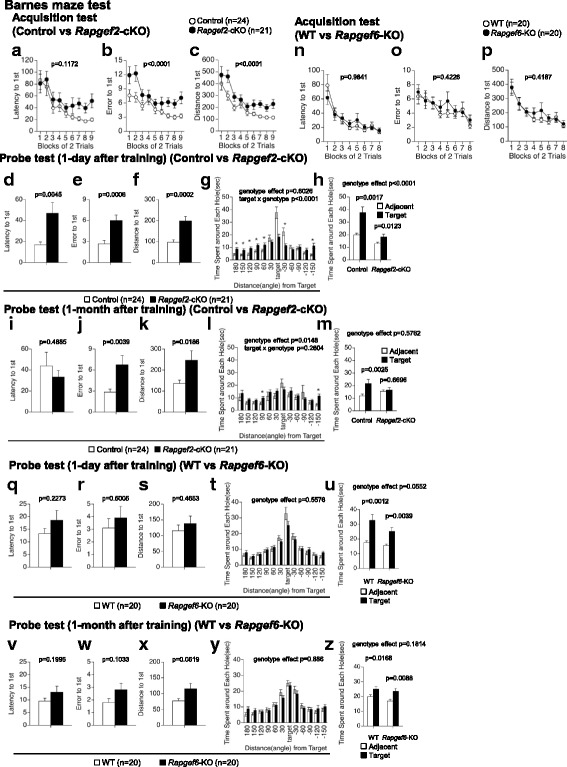
Spatial learning and memory. **a**-**m** Effects of *Rapgef2* deficiency. Data obtained with acquisition tests, where latency (**a**), number of errors made (**b**) and distance traveled (**c**) before acquisition of a target were determined in each block of 2 trials, are shown. Data obtained with probe test performed 1 day after training, where latency (**d**), number of errors made (**e**), and distance traveled (**f**) before acquisition of the target were determined, time spent around each hole whose locations are indicated as “Distance from Target” in angle were determined (**g**), and time spent around the target and its adjacent holes was calculated (**h**), are shown. Data obtained with probe test performed 1 month after training, where latency (**i**), number of errors made (**j**), and distance traveled (**k**) before acquisition of the target were determined, time spent around each hole whose locations are indicated as “Distance from Target” in angle were determined (**l**), and time spent around the target and its adjacent holes was calculated (**m**), are shown. **n**-**z** Effects of *Rapgef6* deficiency. Data obtained with acquisition test (**n**-**p**), probe test 1 day after training (**q**-**u**), and probe test 1 month after training (**v**-**z**), all of which were performed with methods equivalent to those for examining effects of *Rapgef2* deficiency, are shown. *, *p* < 0.05

Effects of *Rapgef2* or *Rapgef6* deficiency on the spatial reference memory were assessed by the Barnes circular maze test. During acquisition tests, *Rapgef2*-cKO mice needed longer time (Fig. 6a, F_1,43_ = 2.556, *p* = 0.1172), made more search errors (Fig. 6b, F_1,43_ = 18.985, *p* < 0.0001) and traveled longer distances before reaching the correct target hole (Fig. 6c, F_1,43_ = 18.746, *p* < 0.0001), indicating that the learning performance was lower in* Rapgef2*-cKO mice. The probe trials using the maze from which an escape box was omitted were performed 1 day and 1 month after training. In the 1-day probe tests, *Rapgef2-*cKO mice needed longer time (Fig. 6d, F_1,43_ = 8.983, *p* = 0.0045), made a larger number of errors (Fig. 6e, F_1,43_ = 13.605, *p* = 0.0006) and traveled longer distances (Fig. 6f, F_1,43_ = 16.909, *p* = 0.0002) before getting to the correct target hole. Although genotype effect was not evident, a two-way repeated measures ANOVA indicated that there was a statistically significant interaction between the time spent around each hole and *Rapgef2* genotype (Fig. 6g, genotype effect, F_1,43_ = 0.062, *p* = 0.8026; genotype × target interaction, F_1, 43_ = 9.572, *p* < 0.0001). The accuracy of spatial memory in *Rapgef2*-cKO mice seemed to be worse than that of control mice (Fig. 6h, control mice, *t* = 3.552, df = 23, *p* = 0.0017; *Rapgef2*-cKO mice, *t* = 2.752, df = 20, *p* = 0.0123, target vs. adjacent holes, paired *t*-test; genotype effect, F_1,43_ = 23.594, *p* < 0.0001; genotype × target interaction, F_1, 43_ = 4.897, *p* = 0.0323, two-way repeated measures ANOVA). In the 1-month probe tests, although the latency to reach the correct target hole was not significantly affected (Fig. 6i, F_1,42_ = 0.488, *p* = 0.4885), *Rapgef2*-cKO mice made significantly more errors (Fig. 6j, F_1,42_ = 9.308, *p* = 0.0039) and traveled longer distance (Fig. 6k, F_1,42_ = 5.991, *p* = 0.0186) before arriving to the target hole. The *Rapgef2* genotype significantly affected the time spent around the hole (Fig. 6l, genotype effect, F_1,42_ = 6.468, *p* = 0.0148). The accuracy of spatial memory seemed to be worse in *Rapgef2*-cKO mice (Fig. 6m, control mice, *t* = 3.392, df = 23, *p* = 0.0025, *Rapgef2*-cKO mice, *t* = 0.433, df = 19, *p* = 0.6696, target holes vs. adjacent holes, paired *t*-test; genotype effect, F_1,43_ = 0.317, *p* = 0.5762, genotype × target interaction, F_1,43_ = 5.445, *p* = 0.0244, two-way repeated measures ANOVA). In striking contrast, *Rapgef6*-KO mice did not exhibit any deficiency in the Barnes circular maze test. There were no significant differences in the latency to find the target hole (Fig. 6n, F_1,38_ = 0.0004, *p* = 0.9841), the number of search errors made (Fig. 6o, F_1,38_ = 0.657, *p* = 0.4228), and the distance to reach the target hole (Fig. 6p, F_1,38_ = 0.668, *p* = 0.4187) during acquisition. In the probe tests performed at both 1 day and 1 month after training, *Rapgef6*-KO mice did not show any significant difference compared to wild-type mice in the latency (1-day probe test, Fig. 6q, F_1,38_ = 1.506, *p* = 0.2273; 1-month probe test, Fig. 6v, F_1,38_ = 1.705, *p* = 0.1995), the number of search errors (1-day probe test, Fig. 6r, F_1,38_ = 0.463, *p* = 0.5005; 1-month probe test, Fig. 6w, F_1,38_ = 2.786, *p* = 0.1033) and the distance (1-day probe test, Fig. 6s, F_1,38_ = 0.544, *p* = 0.4653; 1-month probe test, Fig. 6x, F_1,38_ = 4.029, *p* = 0.0519). No significant genotype effects were detected in the time spent around each holes (1-day probe test, Fig. 6t, genotype effect, F_1,38_ = 0.35, *p* = 0.5576; 1-month probe test, Fig. 6y, genotype effect, F_1,38_ = 0.021, *p* = 0.886) and the accuracy of spatial memory (1-day probe test, Fig. 6u, genotype effect, F_1,38_ = 0.3912, *p* = 0.0552; control mice, *t* = 3.79, df = 29, *p* = 0.0012; *Rapgef6*-KO mice, *t* = 3.282, df = 19, *p* = 0.0039, target vs. adjacent holes, paired *t*-test; 1-month probe test, Fig. 6z, genotype effect, F_1,38_ = 1.853, *p* = 0.1814; control mice, *t* = 2.620, df = 19, *p* = 0.0168; *Rapgef6*-KO mice, *t* = 2.920, df = 19, *p* = 0.0088, target vs. adjacent holes, paired *t*-test).

**Fig. 7:**
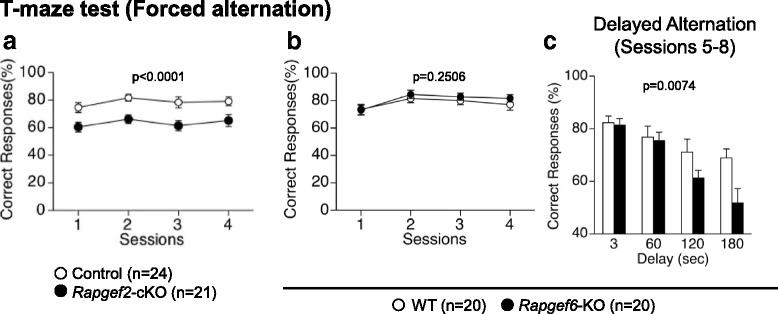
T-maze test. Effects of *Rapgef2* (**a**) or* Rapgef6* (**b**, **c**) deficiency were examined

We performed the T-maze test to assess the genotype effects on the working memory (Fig. 7). We employed a protocol for a forced alternation task test with food deprivation (for examining the effects of *Rapgef2* deficiency) and that for a forced alternation task test without food deprivation (for examining the effects of *Rapgef6* deficiency). These protocols are being widely used for the assessment of the working memory defects even though the level of exploring motivation, such as that associated with the presence or absence of reinforcement, is different among these two protocols. In both protocols, mice were subjected to four consecutive sessions to remember the locations of the arms that were previously visited. *Rapgef2*-cKO mice had a significantly decreased percentage of correct answers in the sessions 1 through 4 compared to control mice (Fig. 7a, F_1,43_ = 30.696, *p* < 0.0001). In contrast, we failed to detect the effects of *Rapgef6* deficiency on the percentage of correct answers in sessions 1 through 4 (Fig. 7b, F_1,38_ = 1.361, *p* = 0.2506). Thus, we performed the delayed alternation task test, which successfully detected a significant decrease in the percentage of correct answering in *Rapgef6*-KO mice (Fig. 7c, F_1,38_ = 7.996, *p* = 0.0074).

### Effects on fear-conditioned memory

**Fig. 8:**
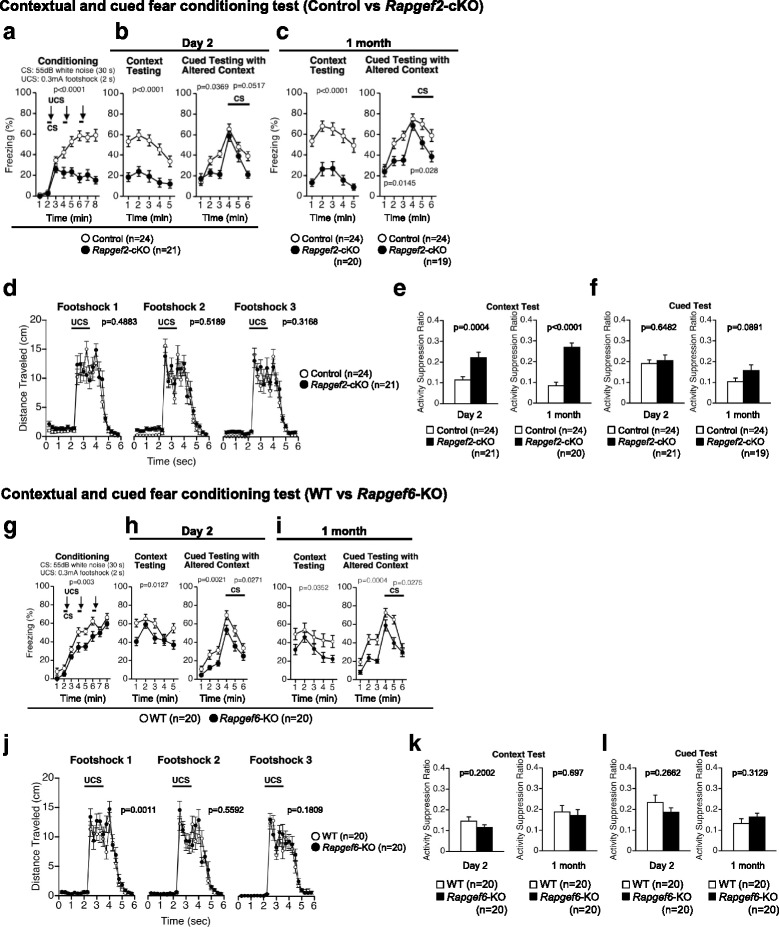
Contextual and cued fear conditioning test. **a**-**f** Effects of *Rapgef2* deficiency. Conditioned stimulus (CS; 55 dB white noise for 30 s) and aversive unconditioned stimulus (UCS; 0.3 mA foot shock for 2 s) were given during the indicated periods. Percentages of freezing time during the conditioning phase (**a**), context testing and cued testing with altered context on day 2 (**b**), and context testing and cued testing with altered context performed 1 month after the last conditioning (**c**) were determined. Distance traveled in each time window of the conditioning phase was determined (**d**). Activity suppression ratios were determined for the context test (**e**) and cued test (**f**). **g**-**l** Effects of *Rapgef6* deficiency. The tests examining the effects of *Rapgef6* deficiency were performed as in **a**-**f**, and percentages of freezing time (**g**-**i**), distance traveled in the conditioning phase (**j**), and activity suppression ratios (**k**, **l**) were determined

Fear-conditioned memory was assessed by a contextual and cued fear conditioning test. In *Rapgef2*-cKO mice, the percentage of the time of freezing caused by the foot-shock was significantly reduced during the conditioning phase (Fig. 8a, F_1,43_ = 32.949, *p* < 0.0001). However, the distance traveled during electrical foot shocks was not significantly impacted by *Rapgef2* genotype (Fig. 8d, foot shock 1, F_1,43_ = 10.489, *p* = 0.4883; foot shock 2, F_1,43_ = 0.423, *p* = 0.5189; foot shock 3, F_1,43_ = 1.026, *p* = 0.3168), suggesting that *Rapgef2* deficiency had no effect on the sensitivity to the electric foot-shock. In the context testing performed at day 2 (1 day after the last conditioning), the percentage of the freezing time was significantly decreased in *Rapgef2*-cKO mice compared to control mice (Fig. 8b, *left*; F_1,43_ = 36.049, *p* < 0.0001). Given the possibility that the percentage of freezing time could have been underestimated in *Rapgef2*-cKO mice due to their hyperlocomotion phenotype, we used an activity suppression ratio as a secondary index of fear in order to control baseline activity [38, 39]. In *Rapgef2*-cKO mice, the calculated activity suppression ratio was significantly increased (Fig. 8e, *left*, F_1,43_ = 15.048, *p* = 0.0004). The cued testing with an altered context was then performed. Before the conditioned stimulus (CS; 55 dB white noise) was given, the percentage of the freezing time were significantly reduced in *Rapgef2*-cKO mice compared to that in control mice (Fig. 8b, *right*, during pre-CSperiod, F_1,43_ = 4.641, *p* = 0.0369). There was an observed tendency that the percentage of the freezing time in *Rapgef2*-cKO mice was reduced during stimulation with the CS (Fig. 8b, *right*, during CS, F_1,43_ = 4.003, *p* = 0.0517). However, the activity suppression ratio during the CS was not significantly affected by *Rapgef2* genotype (Fig. 8f, *left*, F_1,43_ = 0.211, *p* = 0.6482). Similar tests conducted 1 month after the last conditioning revealed a reduction in the percentage of the freezing time in *Rapgef2*-cKO mice (Fig. 8c). In the context test, *Rapgef2*-cKO mice exhibited a significant decrease in the percentage of freezing (Fig. 8c, *left*, F_1,42_ = 36.559, *p* < 0.0001) as well as a significant increase in the activity suppuration ratio (Fig. 8e, *right*, F_1,42_ = 46.445, *p* < 0.0001). In the cued test, the percentage of the freezing time was significantly reduced in *Rapgef2*-cKO mice regardless of the presence or absence of the CS (Fig. 8c, during pre-CS period, F_1,41_ = 6.521, *p* = 0.0145; during CS, F_1,41_ = 5.188, *p* = 0.028), but the activity suppression was not statistically affected by *Rapgef2* genotype (Fig. 8f, *right*, F_1,41_ = 3.032, *p* = 0.0891). Next, similar experiments were carried out with *Rapgef6*-KO mice. In the conditioning phase, the percentage of the time of freezing caused by the foot-shock was significantly reduced in *Rapgef6*-KO mice (Fig. 8g, F_1,38_ = 10.046, *p* = 0.003). There was a significant difference in distance traveled during first electrical foot shock between *Rapgef6*-KO and wild-type mice (Fig. 8j, foot shock 1, F_1,38_ = 12.428, *p* = 0.0011; foot shock 2, F_1,38_ = 0.347, *p* = 0.5592; foot shock 3, F_1,38_ = 1.858, *p* = 0.1809). In the context testing performed at day 2 (1 day after the last conditioning), the percentage of the freezing time was decreased in *Rapgef6*-KO mice compared to wild-type mice (Fig. 8h, left; F_1,38_ = 6.837, *p* = 0.0127). However, the activity suppression ratio was not significantly increased, suggesting underestimation of the percentage of the freezing time probably due to the hyperlocomotion phenotype (Fig. 8k, *left*, F_1,38_ = 1.699, *p* = 0.2002). The cued testing with an altered context was then performed. Before the conditioned stimulus (CS; 55 dB white noise) was given, the percentage of the freezing time was significantly reduced in *Rapgef6*-KO mice compared to that in wild-type mice (Fig. 8h, *right*, during pre-CS period, F_1,38_ = 10.947, *p* = 0.0021). Further, in *Rapgef6*-KO mice, we observed a reduced percentage of the freezing time during stimulation with the CS (Fig. 8h, right, during CS, F_1,38_ = 5.287, *p* = 0.0271). However, the activity suppression ratio during the CS was not significantly affected by the *Rapgerf6* genotype (Fig. 8l, *left*, F_1,38_ = 1.274, *p* = 0.2662). Similar tests conducted 1 month after the last conditioning showed similar reduction in the percentage of the freezing time (Fig. 8i). In the context test, *Rapgef6*-KO mice exhibited a decrease in the percentage of freezing (Fig. 8i, *left*, F_1,38_ = 4.772, *p* = 0.0352), but the activity suppression was not statistically affected by the *Rapgef6* genotype (Fig. 8k, *right*, F_1,38_ = 0.154, *p* = 0.697). In the cued test, the percentage of the freezing time was significantly reduced in *Rapgef6*-KO mice regardless of the presence or absence of the CS (Fig. 8i, during pre-CS period, F_1,38_ = 15.314, *p* = 0.0004; during CS, F_1,38_ = 5.254, *p* = 0.0275), but the activity suppression was not statistically affected by *Rapgef6* genotype (Fig. 8l, *right*, F_1,38_ = 1.046, *p* = 0.3129).

## Discussion and conclusions

In this study, we performed a comprehensive battery of behavioral tests using mice deficient in *Rapgef2* or *Rapgef6* to gain insights into the role of Rapgef2 and Rapgef6 in higher brain functions in mammals. Results obtained in this study are summarized in Table 1. Our results revealed that *Rapgef2* deficiency had more drastic impact on the mice behavior than *Rapgef6* deficiency. This could be partly attributed to the structural abnormalities evident in the brains of *Rapgef2*-cKO mice, including the interruption of pyramidal cells in the hippocampal CA1 region, CC agenesis, ECM, and enlarged lateral ventricles [24–26]. Indeed, such alterations in the brain structure have been implicated in a variety of disorders of higher brain functions. For instance, the hippocampus is important for learning and memory recalling [40], and the interhemispheric connections, including those with the CC, play an important role in coordination of the activities of each hemisphere [41], thereby contributing to the regulation of a variety of brain functions, such as memory fix or maintenance, memory recall, and spatial memory formation [42–45].

**Table 1:** Phenotype of Rapgef2-cKO and Rapgef6-KO mice in comprehensive behavioral test battery

Task	Test	Measure	*Rapgef2*-cKO	*Rapgef6*-KO	Statistical analysis		Figure
			(vs. Non-cKO)	(vs. WT)	*Rapgef2*-cKO	*Rapgef6*-KO	
Physical characteristics	Body weight	Weight (g)	n.s.	– –	F_1,43_ = 1.745, *p* = 0.1935	F_1,38_ = 12.541, *p* = 0.0011	Fig. 1a, e
	Body temperature	Temperature (°C)	n.s.	n.s.	F_1,43_ = 1.991, *p* = 0.1654	F_1,38_ = 4.036, *p* = 0.0517	Fig. 1b, f
	Grip strength (N)	Strength (N)	n.s.	n.s.	F_1,43_ = 1.149, *p* = 0.2897	F_1,38_ = 0.062, *p* = 0.8053	Fig. 1c, g
	Wire hang latency (s)	Latency (s)	– –	n.s.	F_1,43_ = 16.785, *p* = 0.0002	F_1,38_ = 0.04, *p* = 0.8425	Fig. 1d, h
Motor coordination	Rotarod	Latency to fall (s)	n.s.	n.s.	F_1,43_ = 1.251, *p* = 0.2695	F_1,38_ = 0.708, *p* = 0.4052	Fig. 1i, j
Pain sensitivity	Hot plate	Latency (s)	–	n.s.	F_1,43_ = 5.043, *p* = 0.0299	F_1,38_ = 1.045, *p* = 0.3131	Fig. 1k, l
Exploratory locomotion	Open field test	Distance traveled (cm)	+ + +	+ (initial phase)	F_1,43_ = 36.348, *p* < 0.0001	F_1,38_ = 0.03, *p* = 0.8638	Fig. 2a, c
Anxiety-like behavior	Open field test	Center time (s)	+ + +	+ (initial phase)	F_1,43_ = 19.658, *p* < 0.0001	F_1,38_ = 0.002, *p* = 0.9656	Fig. 2b, d
	Light/dark transition test	Number of transitions	n.s.	n.s.	F_1,43_ = 0.735, *p* = 0.3961	F_1,38_ = 0.395, *p* = 0.5335	Fig. 2e, g
		Latency to light chamber (s)	n.s.	n.s.	F_1,43_ = 0.501, *p* = 0.4827	F_1,38_ = 0.669, *p* = 0.4187	Fig. 2f, h
	Elevated plus maze test	Entries into open arms (%)	+ +	n.s.	F_1,42_ = 8.453, *p* = 0.0058	F_1,38_ = 1.328, *p* = 0.2564	Fig. 2i, k
		Time on open arms (%)	+	n.s.	F_1,42_ = 6.229, *p* = 0.0166	F_1,38_ = 0.095, *p* = 0.7597	Fig. 2j, l
Behavioral despair	Porsolt forced swim test	Immobility (%) on Day 1	n.s.	- (1-7 min)	F_1,43_ = 2.71, *p* = 0.107	F_1,38_ = 1.881, *p* = 0.1783; 1-7 min, F_1,38_ = 5.089, *p* = 0.0299	Fig. 3a, b
		Immobility (%) on Day 2	- (1-3 min), + + (4-10 min)	- (1-2 min)	F_1,43_ = 0.065, *p* = 0.8007; 1-3 min, F_1,43_ = 6.82, *p* = 0.0124; 4-10 min, F_1,43_ = 17.252, *p* = 0.0002	F_1,38_ = 2.952, *p* = 0.0939; 1-2 min, F_1,38_ = 4.865, *p* = 0.0335	Fig. 3a, b
	Tail suspension test	Immobility (%)	+ + (7-10 min)	n.s.	F_1,43_ = 3.569, *p* = 0.0656; 7-10 min, F_1,43_ = 8.574, *p* = 0.0054	F_1,38_ = 2.651, *p* = 0.1118	Fig. 3c, d
Sensorimotor gating	Startle response test	Startle response	–	–	F_1,43_ = 4.129, *p* = 0.0484	F_1,38_ = 6.287, *p* = 0.0166	Fig. 4a, c
	Prepulse inhibition test	PPI (startle stimulus, 110 dB)	n.s.	n.s.	F_1,43_ = 1.161, *p* = 0.2873	F_1,38_ = 1.007, *p* = 0.322	Fig. 4b, d
		PPI (startle stimulus, 120 dB)	n.s.	n.s.	F_1,43_ = 0.293, *p* = 0.5912	F_1,38_ = 0.222, *p* = 0.6404	Fig. 4b, d
Social interaction	Novel environment	Total duration of contacts (s)	n.s.	n.s.	F_1,18_ = 0.048, *p* = 0.8299	F_1,18_ = 0.215, *p* = 0.6484	Fig. 5a, f
		Number of contacts	+ + +	+ +	F_1,18_ = 28.989, *p* < 0.0001	F_1,18_ = 9.088, *p* = 0.0074	Fig. 5b, g
		Total duration of active contacts (s)	+ + +	n.s.	F_1,18_ = 27.519, *p* < 0.0001	F_1,18_ = 4.132, *p* = 0.0571	Fig. 5c, h
		Mean duration of contact (s)	–	–	F_1,18_ = 5.566, *p* = 0.0298	F_1,18_ = 5.719, *p* = 0.0279	Fig. 5d, i
		Distance traveled (cm)	+ + +	+	F_1,18_ = 39.017, *p* < 0.0001	F_1,18_ = 6.421, *p* = 0.0208	Fig. 5e, j
	Home cage	Mean number of particles	n.s.	n.s.	F_1,16_ = 0.743, *p* = 0.4016; light period, F_1,16_ = 0.031, *p* = 0.8628; dark period, F_1,16_ = 1.65, *p* = 0.2173	F_1,18_ = 0.002, *p* = 0.9695; light period, F_1,18_ = 0.438, *p* = 0.5163; dark period, F_1,18_ = 0.143, *p* = 0.7097	Fig. 5k, m
		Activity levels	n.s.	n.s.	F_1,16_ = 0.141, *p* = 0.712; light period, F_1,16_ = 0.928, *p* = 0.3497; dark period, F_1,16_ = 0.546, *p* = 0.4705	F_1,18_ = 0.031, *p* = 0.8633; light period, F_1,18_ = 0.96, *p* = 0.3401; dark period, F_1,18_ = 0.169, *p* = 0.6858	Fig. 5l, n
	Crawley’s version	Time spent around cage (s) on *1st*	– –	n.s.	F_1,43_ = 8.754, *p* = 0.005	F_1,38_ = 1.221, *p* = 0.276	Fig. 5o, p
		Time spent around cage (s) on *2nd*	– –	n.s.	F_1,43_ = 9.539, *p* = 0.0035	F_1,38_ = 0.014, *p* = 0.9074	Fig. 5o, p
Spatial memory	Barnes maze test						
	Acquisition test	Latency to 1st (s)	n.s.	n.s.	F_1,43_ = 2.556, *p* = 0.1172	F_1,38_ = 0.0004, *p* = 0.9841	Fig. 6a, n
		Error to 1st	+ + +	n.s.	F_1,43_ = 18.985, *p* < 0.0001	F_1,38_ = 0.657, *p* = 0.4228	Fig. 6b, o
		Distance to 1st (mm)	+ + +	n.s.	F_1,43_ = 18.746, *p* < 0.0001	F_1,38_ = 0.668, *p* = 0.4187	Fig. 6c, p
	Probe test (1-day after training)	Latency to 1st (s)	+ +	n.s.	F_1,43_ = 8.983, *p* = 0.0045	F_1,38_ = 1.506, *p* = 0.2273	Fig. 6d, q
		Error to 1st	+ +	n.s.	F_1,43_ = 13.605, *p* = 0.0006	F_1,38_ = 0.463, *p* = 0.5005	Fig. 6e, r
		Distance to 1st (mm)	+ +	n.s.	F_1,43_ = 16.909, *p* = 0.0002	F_1,38_ = 0.544, *p* = 0.4653	Fig. 6f, s
		Time spent around each hole (s)	n.s.	n.s.	F_1,43_ = 0.062, *p* = 0.8026	F_1,38_ = 0.35, *p* = 0.5576	Fig. 6g, t
		Time spent around target hole (s)	– – –	n.s.	F_1,43_ = 23.594, *p* < 0.0001	F_1,38_ = 0.3912, *p* = 0.0552	Fig. 6h, u
	Probe test (1-month after training)	Latency to 1st (s)	n.s.	n.s.	F_1,42_ = 0.488, *p* = 0.4885	F_1,38_ = 1.705, *p* = 0.1995	Fig. 6i, v
		Error to 1st	+ +	n.s.	F_1,42_ = 9.308, *p* = 0.0039	F_1,38_ = 2.786, *p* = 0.1033	Fig. 6j, w
		Distance to 1st (mm)	+	n.s.	F_1,42_ = 5.991, *p* = 0.0186	F_1,38_ = 4.029, *p* = 0.0519	Fig. 6k, x
		Time spent around each hole (s)	n.s.	n.s.	F_1,42_ = 6.468, *p* = 0.0148	F_1,38_ = 0.021, *p* = 0.886	Fig. 6l, y
		Time spent around target hole (s)	n.s.	n.s.	F_1,43_ = 0.317, *p* = 0.5762	F_1,38_ = 1.853, *p* = 0.1814	Fig. 6m, z
Working memory	T-maze test	Correct responses (%)	– – –	- - (delayed alternation)	F_1,43_ = 30.696, *p* < 0.0001	Session 1-4, F_1,38_ = 1.361, *p* = 0.2506 Session 5-8, F_1,38_ = 7.996, *p* = 0.0074	Fig. 7a, c
Cued and contextual fear conditioning	Fear conditioning test						
	Conditioning	Freezing (%)	– – –	– –	F_1,43_ = 32.949, *p* < 0.0001	F_1,38_ = 10.046, *p* = 0.003	Fig. 8a, g
	Context test 1 day after conditioning	Freezing (%)	– – –	–	F_1,43_ = 36.049, *p* < 0.0001	F_1,38_ = 6.837, *p* = 0.0127	Fig. 8b, h
	Cued test 1 day after conditioning (pre-CS)	Freezing (%)	–	– –	F_1,43_ = 4.641, *p* = 0.0369	F_1,38_ = 10.947, *p* = 0.0021	Fig. 8b, h
	Cued test 1 day after conditioning (CS)	Freezing (%)	n.s.	–	F_1,43_ = 4.003, *p* = 0.0517	F_1,38_ = 5.287, *p* = 0.0271	Fig. 8b, h
	Context test 1 month after conditioning	Freezing (%)	– – –	–	F_1,42_ = 36.559, *p* < 0.0001	F_1,38_ = 4.772, *p* = 0.0352	Fig. 8c, i
	Cued test 1 month after conditioning (pre-CS)	Freezing (%)	–	– –	F_1,41_ = 6.521, *p* = 0.0145	F_1,38_ = 15.314, *p* = 0.0004	Fig. 8c, i
	Cued test 1 month after conditioning (CS)	Freezing (%)	–	–	F_1,41_ = 5.188, *p* = 0.028	F_1,38_ = 5.254, *p* = 0.0275	Fig. 8c, i
	Fear conditioning test (foot shock 1)	Distance traveled (cm)	n.s.	+ +	F_1,43_ = 10.489, *p* = 0.4883	F_1,38_ = 12.428, *p* = 0.0011	Fig. 8d, j
	Fear conditioning test (foot shock 2)	Distance traveled (cm)	n.s.	n.s.	F_1,43_ = 0.423, *p* = 0.5189	F_1,38_ = 0.347, *p* = 0.5592	Fig. 8d, j
	Fear conditioning test (foot shock 3)	Distance traveled (cm)	n.s.	n.s.	F_1,43_ = 1.026, *p* = 0.3168	F_1,38_ = 1.858, *p* = 0.1809	Fig. 8d, j
	Context test 1 day after conditioning	Activity suppression ratio	+ +	n.s.	F_1,43_ = 15.048, *p* = 0.0004	F_1,38_ = 1.699, *p* = 0.2002	Fig. 8e, k
	Context test 1 month after conditioning	Activity suppression ratio	+ + +	n.s.	F_1,42_ = 46.445, *p* < 0.0001	F_1,38_ = 0.154, *p* = 0.697	Fig. 8e, k
	Cued test 1 day after conditioning (CS)	Activity suppression ratio	n.s.	n.s.	F_1,43_ = 0.211, *p* = 0.6482	F_1,38_ = 1.274, *p* = 0.2662	Fig. 8f, l
	Cued test 1 month after conditioning (CS)	Activity suppression ratio	n.s.	n.s.	F_1,41_ = 3.032, *p* = 0.0891	F_1,38_ = 1.046, *p* = 0.3129	Fig. 8f, l

*n.s*. no significance

Nominal significance: +/− *p* < 0.05, + +/− − *p* < 0.01, + + +/− − − *p* < 0.001

The open field test clearly detected hyperlocomotion phenotype in *Rapgef2*-cKO mice (Fig. 2a, b). This hyperlocomotion phenotype in *Rapgef2* mutant mice seemed not to be affected by aging because it was detected both in younger and elder *Rapgef2*-cKO mice. In contrast, although *Rapgef6*-KO mice did not clearly exhibit difference in the total distance traveled, the *Rapgef6* genotype significantly affected these indices in a time-dependent manner (Fig. 2c, genotype × time interaction, F_23,874_ = 2.350, *p* = 0.0004). Even though the degree of the phenotypic changes in the locomotion was greater in *Rapgef2*-cKO mice than in *Rapgef*6-KO mice, the hyperlocomotion phenotypes appeared to be detected also in other tests performed in this study and seemed to affect the outputs of the behavioral tests. Thus, their hyperlocomotion phenotypes needed to be considered as confounding factors that should be paid particular attention upon the interpretation of data obtained here.

In *Rapgef2*-cKO mice, we detected depression-like phenotypes in the tail suspension test (Fig. 3c), where depression-like behavior was judged as increased immobility. *Rapgef2*-cKO mice exhibited depression-like phenotypes also in the porsolt forced swim test on day2, where immobility that could be associated with depression was increased in the 4-10 min windows (Fig. 3a, *right*). On the other hand, a decrease in the immobility detected in the 1-3 min windows seemed to be associated with increased locomotor activity and/or with impaired memory of *Rapgef2*-cKO mice as discussed later. However, in *Rapgef6*-KO mice, we failed to detect phenotypic alterations associated with depression using the tail suspension test (Fig. 3d). In the porsolt forced swim test, *Rapgef6*-KO mice exhibited decreased immobility that could be associated with their hyperlocomotion phenotypes (Fig. 3b). Reduction in anxiety-like behavior was detected in *Rapgef2*-cKO mice by the open field test, where the time spent in the arena center was significantly increased (Fig. 2b). Reduced anxiety-like behavior in *Rapgef2*-cKO mice was also evident in the elevated plus maze test, where the percentages of entries into the open arms and the time spent in the open arms were significantly increased (Fig. 2i, j), further supporting the notion obtained by the open field test. On the other hand, it might be hard to conclude that *Rapgef6* deficiency affected anxiety-like behavior because the elevated plus maze test and the light/dark transition test failed to detect changes associated with the *Rapgef6* genotype (Fig. 2g, h, k, l) although *Rapgef6*-KO mice exhibited mild changes in the time spent in the arena center in the early and late phases of the open field test (Fig. 2c, d).

To examine the effects on sociability, we performed social interaction tests (Fig. 5). Overall, social interaction tests with a novel environment (Fig. 5a-j) and those in a home cage (Fig. 5k-n) did not reveal significant genotype effects except for some indices. For the indices showing genotype dependency such as number of contacts (Fig. 5b, g) and total duration of active contacts (Fig. 5c, h), it was difficult to rule out the possibility that the increase associated with the genotypes was simply caused by the hyperlocomotion phenotypes solely based on the data obtained from these social interaction tests. However, Crawley’s version of sociability tests demonstrated that *Rapgef2*-cKO mice preferred to stay around the stranger cage than control mice although the control animals that were compared to *Rapgef2*-cKO mice seemed slightly poor at discriminating an empty cage and a stranger-mouse-containing cage (Fig. 5o). These results indicated that the test detected increased sociability in *Rapgef2*-cKOmice (Fig. 5o). In contrast, such trends were not obviously detected with *Rapgef6*-KO mice (Fig. 5p). These results indicated that only* Rapgef2* genotype affected sociability.

In the current study, our results demonstrated that *Rapgef2*-cKO mice and *Rapgef6*-KO mice exhibited defects in the learning and memory tasks. Data obtained by the T-maze test suggested impaired working memory in *Rapgef2*-cKO mice (Fig. 7a). Working memory defects were also detected in *Rapgef6*-KO mice even though the protocol employed was slightly different from that used for *Rapgef2*-cKO mice (Fig. 7b, c). Data obtained by the Barnes circular maze test suggested that the acquisition and retention of the spatial reference memory were impaired in *Rapgef2*-cKO mice (Fig. 6). On the other hand, *Rapgef6*-KO mice failed to show an obvious abnormality in this test, consistent with the previous report using the same *Rapgef6* mutant strain [27]. Taken together, these results suggested that *Rapgef2* deficiency, rather than *Rapgef6* deficiency, decreases the learning ability. We also performed the contextual and cued fear conditioning test (Fig. 8). In this test, *Rapgef2*-cKO mice exhibited altered performance during the conditioning phase (Fig. 8a), which may reflect their reduced learning ability and/or hyperlocomotion phenotype. Their altered learning ability and hyperlocomotion phenotypes also seem to affect the outcomes of the tests performed on day 2 and 1 month after conditioning, where *Rapgef2*-cKO mice again exhibited reduced freezing in both context testing and cued testing with altered context (Fig. 8b, c). However, the activity suppression ratio indicates that *Rapgef2* deficiency tended to reduce the performance more significantly 1 month after conditioning than on day 2 (Fig. 8e, f). Therefore, we cannot rule out the possibility that long-term memory maintenance might be impaired in *Rapgef2*-cKO mice. To clarify these points, further studies are required by employing another experimental system, such as that where *Rapgef2* is inactivated after conditioning. In *Rapgef6*-KO mice, we also detected similar tendency, such as reduced freezing (Fig. 8g-i). However, the activity suppression ratio indicated that reduced freezing in *Rapgef6*-KO mice was caused as a result of their hyperlocomotive tendencies rather than by memory or learning defects (Fig. 8k, l). Contrary to our current results, previous studies performed at a different facility using the same *Rapgef6*-KO mouse strain concluded that *Rapgef6*-KO mice had deficiencies in context-dependent memory [27]. However, in that study, Levy et al.*,* did not calculate activity suppression ratio and did not consider the hyperlocomotion phenotypes of *Rapgef6*-KO mice [27]. Therefore, we speculate that this discrepancy could be possibly attributed to the underestimation of the impact of the hyperlocomotion phenotypes on the outputs and/or that the differences in experimental facilities and experimenters. Impairment in learning and memory also seemed to affect the outputs of the porsolt forced swim test, in which both *Rapgef2*-cKO mice and *Rapgef6*-KO mice exhibited reduced immobility during the initial phases at day2 (Fig. 3a, b). Although their hyperlocomotion phenotypes could again affect the outputs of this test, another possibility is that *Rapgef2*-cKO mice and *Rapgef6*-KO mice failed to memorize the previous fear experience due to the defects in learning and memory.

*Rapgef2* and *Rapgef6* were implicated in the etiology of schizophrenia by genome-wide association study (GWAS) of patients with the schizophrenic disorder [28, 29]. Therefore, we investigated whether the observed behavioral characteristics of *Rapgef2*-cKO and *Rapgef6*-KO mice were relevant to those of symptomatic schizophrenia patients. For instance, hyperlocomotion phenotypes in *Rapgef2*-cKO mice and *Rapgef6*-KO mice seemed to be analogous to psychomotor agitation evident in schizophrenia patients [46]. Additionally, mice models with psychomotor agitation-like phenotypes, such as those produced by psychostimulant administration, are recognized as a model for schizophrenia [47, 48]. T-maze alternation memory task and Barnes maze indicated a working memory defect and a spatial memory defect, respectively, in *Rapgef2*-cKO mice and *Rapgef6*-KO mice. These defects were reported to be a phenotype of schizophrenia-relevant behavior [47]. However, as discussed above, *Rapgef2*-cKO mice exhibited increased sociability, which seems opposite to the widely accepted concept that reduced sociability recapitulated in experimental animals is a model of social withdrawal observed as a negative symptom of schizophrenia. These suggest that behavioral abnormalities in *Rapgef2*-cKO mice and *Rapgef6*-KO mice recapitulate not only those of a subpopulation of schizophrenia but also those seen in other mental disorder patients.

Rap1 and Rap2 were reported to be involved in the regulation of higher brain functions, such as fear learning and spatial learning [10, 12]. Previous studies focused on Rap proteins as molecules coupling cAMP signaling to the signaling involving extracellular signal regulated kinase (ERK) 1 and ERK2, which regulates excitability, synaptic plasticity, learning and memory [49]. In such cAMP-dependent Rap activation, Rapgef3 and Rapgef4 (also known as Epac1 and Epac2, respectively) have been biochemically characterized as cAMP-dependent Rap-specific GEFs and shown to be involved in spatial learning and social interaction [16, 50, 51]. In contrast to these 2 cAMP-responsive Rap GEFs, Rapgef2 and Rapgef6 cannot be activated by cAMP as we and others previously reported [18–20, 22], and thus they have a role other than transmitting cAMP signaling to Rap activation. In this regard, our study expands our knowledge on the Rap signaling in the regulation of higher brain functions.

To the best of our knowledge, this is the first report that demonstrates that Rapgef2 is involved in regulation of locomotion and working memory. Further, we observed that *Rapgef2* deficiency produced more pronounced defects in the higher brain functions although both Rapgef2 and Rapgef6 share structural and functional similarities. Considering that Rapgef2 and Rapgef6 are abundantly expressed in the cortex [26, 27], they may play an important role in prefrontal cortex-dependent working memory. Further studies at the cellular and molecular levels are required to clarify the mechanisms of Rapgef2- and/or Rapgef6-mediated regulation of the activities of neurons, which may help in understanding the role of *Rapgef2* and *Rapgef6* in the regulation of higher brain functions and the impact of their deficiencies in the development of mental disorders.

## Methods

### Animals and experimental design

Mice used in this study had been backcrossed over 11 times to the C57BL/6 J strain. *Rapgef2*-cKO (*Rapgef2*^flox/flox^;*Emx1*^cre/+^) mice were prepared by mating *Rapgef2*^flox/flox^mice with *Emx*^Cre/+^ mice [24]. *Rapgef6*-KO mice were generated as detailed previously [35]. Behavioral tests were performed with male mice that were at least 14 weeks old. The ages of the mice at the time of each experiment are listed in Tables 2 and 3. In each strain, we run the experiments with a single batch. Mice were group housed in a room with a 12 h light/dark cycle (lights on at 7:00 am) with free access to food and water expect for the period during which the T-maze test was being conducted. Room temperature was kept at 23 ± 2 °C. Behavioral testing was performed between 9:00 am and 6:00 pm. After tests, all apparatus used was cleaned with diluted hypochlorite solution or 70% ethanol to prevent a bias from olfactory cues. To minimize the effects of previous tests on the subsequent tests, we performed the behavioral test battery in the following order: general health and neurological screens, light/dark transition test, open field test, elevated plus maze, hot plate test, social interaction test in a novel environment, rotarod test, Crawley’s sociability and preference for social novelty test, startle response/prepulse inhibition test, Porsolt forced swim test, Barnes maze test, T-maze test, tail suspension test, fear conditioning test, and social interaction test in a home cage. Behavioral tests were performed at intervals of at least 1 day. The use and care of the animals were reviewed and approved by the Institutional Animal Care and Use Committee of Kobe University and that of Fujita Health University. Raw data of the behavioral tests are available at the mouse phenotype database (http://www.mouse-phenotype.org).

**Table 2:** Comprehensive behavioral test battery of *Rapgef2*-cKO mice

Test	Age (w)
1. General health	17–23
2. Light/dark transition	17–24
3. Open field	18–24
4. Elevated plus-maze	18–24
5. Hot plate	19–25
6. Social Interaction (novel environment)	19–25
7. Rotarod	19–26
8. Social Interaction (Crawley version)	22–29
9. Prepulse inhibition	23–30
10. Porsolt forced swim	24–30
11. Barnes maze (training)	42–52
12. Barnes maze (probe test (24 h))	46–52
13. Barnes maze (probe test (1 month))	51–57
14. T-maze (forced alternation with fasting)	60–66
15. T-maze (left-right discrimination)	61–69
16. Tail suspension test	63–69
17. Fear conditioning test (Day 1)	63–70
18. Fear conditioning test (Day 2)	64–70
19. Fear conditioning test (Day 31)	68–74
20. Social Interaction (home cage)	69–76
21. Open field	72–78

*Age (w)* age in weeks of mice at the beginning of each test listed

**Table 3:** Comprehensive behavioral test battery of *Rapgef6*-KO mice

Test	Age (w)
1. General health	14–16
2. Rotarod	20–22
3. Hot plate	18–19
4. Open field	15–17
5. Light/dark transition	15–16
6. Elevated plus-maze	17–19
7. Porsolt forced swim	32–34
8. Tail suspension test	41–42
9. Prepulse inhibition	32–33
10. Social Interaction (novel environment)	19–20
11. Social Interaction (home cage)	59–62
12. Social Interaction (Crawley version)	31–33
13. Barnes maze (training)	32–36
14. Barnes maze (probe test (24 h))	34–36
15. Barnes maze (probe test (1 month))	39–40
16. T-maze (forced alternation without fasting)	39–42
17. Fear conditioning test (Day 1)	41–43
18. Fear conditioning test (Day 2)	41–43
19. Fear conditioning test (Day 36)	46–48

*Age (w)* age in weeks of mice at the beginning of each test listed

### Behavioral tests

#### General health and neurological screens

The presence of whiskers or bald hair patches was recorded. The righting, whisker touch, and ear twitch reflexes were also evaluated. Body weight and rectal temperature were measured. Neuromuscular strength was tested with the grip strength test and wire hang test. Grip strength was measured by a grip strength meter (O’HARA & Co., Tokyo, Japan). Mice were grasped a wire grid by the forelimbs and pulled backward until they release it. The peak force was recorded in Newtons (N). Each mouse was tested three times, and the greatest value obtained was used for further data analyses. In the wire hang test, a mouse was placed on the wire mesh at the top of apparatus (O’HARA & Co., Tokyo, Japan), and the wire mesh was then gently turned inside out. The mouse gripped the wire in order not to fall off, and the latency to fall was recorded with 60-s cut-off time. A one-way ANOVA was used to determine *p* values for genotype effects.

### Rotarod test

An accelerating rotarod (UGO Basile, Comerio, VA, Italy) was used to test the motor coordination and balance of mice. A mouse was placed on a rotating drum (3 cm diameter) and the speed of the rotarod was accelerated from 4 to 40 rpm in 5 min. The animals went through three trials in a day on 2 consecutive days. The length of the period that a mouse was able to maintain its balance on the rod was determined. A two-way repeated measures ANOVA was used to determine *p* values for genotype effects.

### Hot plate test

To evaluate the sensitivity to painful stimuli, a mouse was placed on a hot plate (Columbus Instruments, OH, USA) at 55.0 (± 0.1)°C. The latency to the first fore-paw or hind-paw response, defined by either a foot shake or a paw lick, was recorded with a 15 s cut-off time. A one-way ANOVA was used to determine *p* value for genotype effect.

### Open field test

Locomotor activity was measured using an open field apparatus (40 × 40 × 30 cm; Accuscan Instruments, Columbus, OH, USA) [52]. The test chamber was illuminated at 100 ± 5 lx. A mouse was placed in the corner of the apparatus. Total distance traveled (cm) and time spent in the center area of the open field (20 × 20 cm) were recorded by the VersaMax system. Data were collected for 120 min. A two-way repeated measures ANOVA was used to determine *p* values for genotype effects.

### Light/dark transition test

The light/dark transition test was performed as previously described [52, 53]. The apparatus comprised a box (21 × 42 × 25 cm) that was divided equally into 2 sections with a partition having a door (O’HARA & Co., Tokyo, Japan). One chamber was brightly illuminated (390 ± 5 lx) whereas the other one was kept dark (2 lx). A mouse was placed into the dark chamber and allowed to move freely between the 2 chambers through the door open for 10 min. Total number of transitions and latency to the first enter to the light chamber were recorded using ImageLD software. A one-way ANOVA was used to determine *p* values for genotype effects.

### Elevated plus-maze test

To evaluate anxiety-related behavior, the elevated plus maze test was conducted as previously described [52, 54]. The apparatus consisted of 2 open arms (25 × 5 cm) and 2 arms enclosed with 15 cm high transparent walls, all of which were elevated 55 cm above the floor (O’HARA & Co., Tokyo, Japan). Arms of the same type were located opposite from each other. In order to minimize the chance that animals fell from the apparatus, 3-mm high plexiglas ledges were equipped in the open arms. A mouse was placed in the central square of the maze (5 × 5 cm) so that it faced one of the enclosed arms. Number of entries into the each kind of arms and time spent (sec) in each kind of arms were recorded for 10 min. Percentage of entries into the open arms and that of time spent in the open arms were determined. Data acquisition and analysis were performed automatically using ImageEP software. A one-way ANOVA was used to determine *p* values for genotype effects.

### Porsolt forced swim test

A transparent plastic cylinder (22 cm height × 12 cm diameter), which was filled with water (approximately 23 °C) up to a height of 7.5 cm, was put in a white plastic chamber (O’HARA & Co., Tokyo, Japan). A mouse was placed in the cylinders, and the immobility and the distance traveled were recorded for 10 min by capturing images at 2 frames per second on days1 and 2 [52]. For each pair of successive frames, the amount of area (pixels) in which the mouse moved was measured. If the amount of area was below a certain threshold, which was optimized by human observation of the behavior, the behavior was classified as “immobile”. Immobility lasting for less than 2 s was not included in the analyses. Data acquisition and analysis were performed automatically using ImageTS software. A two-way repeated measures ANOVA was used to determine *p* values for genotype effects. In addition, *p* values for genotype × time interaction (*g* × *t*) were also determined.

### Tail suspension test

Mice were suspended with their tail by adhesive tape at 30 cm above the floor of a white plastic chamber (O’HARA & Co., Tokyo, Japan), and their behavior was recorded for 10 min [53]. Similar to the Porsolt forced swim test, images were captured at 2 frames per second, and immobility was judged by the application program according to a certain threshold, which was optimized by human observation of the behavior. Data acquisition and analysis were performed automatically using ImageTS software. A two-way repeated measures ANOVA was used to determine *p* values for genotype effects. Also, *p* values for genotype × time interaction (*g* × *t*) were determined.

### Startle response/Prepulse inhibition test

A startle reflex measurement system was used (O’HARA & Co., Tokyo, Japan) [49]. A test session began by placing a mouse in a plastic cylinder where it was left undisturbed for 10 min. White noise (40 msec) was used as the startle stimulus for all trial types. The startle response was measured for 400 msec by accelerometer from 100 msec before the onset of the prepulse stimulus. The background noise level in each chamber was 70 dB. A test session consisted of 2 types for startle stimulus-only trials (110 or 120 dB) and 4 types for prepulse inhibition trials (74-110, 78-110, 74-120, and 78-120 dB). The prepulse sound was presented 100 msec before startle stimulus. Six trial types were presented in a pseudo-random order, such that each trial type was presented once within a block. The average inter-trial interval was 15 s (range: 10-20 s). A two-way repeated measures ANOVA was used to determine *p* values for genotype effects.

### Social interaction test in a novel environment

Two mice of identical genotype that had been housed in different cages were placed together in a box (40 × 40 × 30 cm) (O’HARA & Co., Tokyo, Japan), and allowed to explore freely for 10 min the box [52]. Total duration of contacts, total number of contacts, total duration of active contacts, mean duration of a contact, and total distance traveled were measured. If the 2 mice contacted each other and the distance traveled by either mouse was longer than 10 cm, the behavior was considered as an ‘active contact’. Behavior was recorded and analyzed automatically using ImageSI program. Images were captured at 3 frames per second. A one-way ANOVA was used to determine *p* values for genotype effects.

### Social interaction test in a home cage

The system for monitoring social interaction comprised a home cage and a filtered cage top with an infrared video camera (31 × 19 × 30 cm; 25 × 15 × 23.5 cm, inside dimensions) (O’HARA & Co., Tokyo, Japan) [55]. Two mice with the same genotype that had been housed separately were placed together in a home cage. To evaluate social interaction, their social behavior was monitored with a video camera for a week. The occurrence of social interaction was detected by counting the number of particles consisting of the mice as follows: 2 particles indicated that the mice were not in contact whereas 1 particle indicated that 2 mice were in contact. Locomotor activity of the mice was also measured by quantifying the number of pixels that changed between each pair of successive frames while these experiments were performed. Analysis was automatically performed using ImageHA software. A two-way repeated measures ANOVA was used to determine *p* values for genotype effects.

### Crawley’s sociability and social novelty preference test

To investigate the effect of complex genetics on sociability and preference for social novelty, Crawley’s sociability and social novelty preference test was performed as described [36, 37] using an apparatus composed of a three-chambered rectangular box with an infrared video camera (O’HARA & Co., Tokyo, Japan). Each chamber (20 × 40 × 47 cm) was partitioned by a wall with a small square opening (5 × 3 cm) thus allowing mice to access each area. Wire cages (11 cm height × 9 cm diameter, vertical bars 0.5 cm apart), which allowed nose contact between the bars but prevented fighting, were located in the corners of side chambers. “Habituation” session was performed in the apparatus for 10 min. In “sociability” test, an unfamiliar C57BL/6 J male (Stranger 1) was enclosed in the wire cage. The cage containing Stranger 1 was placed in one of the side chambers while the cage placed in the other side chamber was kept empty. A subject mouse was placed in the middle chamber and allowed to move freely for 10 min. To perform “preference” test, after completion of the first 10-min session, a second unfamiliar mouse (Stranger 2) was enclosed in the other wire cage, which had been empty in the first session, and the subject mouse was again placed in the middle chamber and examined as in the first session. In each session, the amount of time spent around each cage was measured. Data acquisition and analysis were performed using ImageCSI. A two-way repeated measures ANOVA was used to determine *p* values for genotype effects whereas paired *t*-test was employed to derive *p* values between the types of the apparatuses. To examine the effects of *Rapgef2* deficiency, after the “habituation” was completed for all individuals to be tested by the following tests, each individual was subjected to the “sociability” test and the “preference” test sequentially. To examine the effects of *Rapgef6* deficiency, each individual was subjected to the “habituation” and then subjected to the “sociability” test and the “preference” test sequentially.

### Barnes maze test

To assess spatial learning, Barnes circular maze was used. The test was conducted on “dry land”, a white circular surface with a diameter of 1.0 m having 12 holes equally spaced around the perimeter (O’HARA & Co., Tokyo, Japan). A black Plexiglas escape box (17 × 13 × 7 cm), which had paper bedding on its bottom, was located under one of the holes. The hole above the escape box represented the target. The spatial location of the target was consistent for a given mouse but randomized across mice subjected to the tests. The maze was rotated daily to prevent a bias caused by olfactory or proximal cues in the maze. The spatial location of the target was unchanged with respect to the visual room cues. During acquisition test, each trial ended when a mouse entered the escape box or after 5 min had elapsed. Mice that failed to find the box were guided to it. Latency and distance to reach the target hole, number of errors (defined by placing mouse’s nose in a hole that did not have an escape box) were recorded using ImageBM software. A one-way ANOVA and two-way repeated measures ANOVA was used to determine *p* values for genotype effects. One day after the last training, a probe trial was performed using the same apparatus, from which the escape box was omitted, to assess the memory based on distal environmental cues. Another probe test was performed 1 month later to evaluate retention of spatial memory. In the probe tests, time spent around the target hole was also recorded by ImageBM software. To judge whether the differences observed were statistically significant, *p* values for genotype effects determined by a one-way ANOVA. For the time spent around each hole, *p* values for genotype effects and those for target × genotype interaction were determined by a two-way repeated measures ANOVA. For the time spent around the target and its adjacent holes, *p* values for genotype effects and for target × genotype interaction were determined by a two-way repeated measures ANOVA, and those between the target and its adjacent holes for each genotype were determined by paired *t*-test.

### T-maze test

To assess working memory, we employed a forced alternation task test with food deprivation (for examining the effects of *Rapgef2* deficiency) and a forced alternation task test without food deprivation (for examining the effects of *Rapgef6* deficiency) using T-maze apparatus (O’HARA & Co., Tokyo, Japan) [55]. To assess the effects of *Rapgef2* deficiency, mice were subjected to the forced alternation task test for 4 days (one session consisting of 10 trials per day; cut-off time, 50 min). In this forced alternation test, before the pre-training, mice to be subjected to the test were deprived of food until their bodyweight was reduced to 80-85% of their initial bodyweight. On the other hand, to assess the effects of *Rapgef6* deficiency, mice were tested with another protocol for the forced alternation task test from which fasting before the test was omitted. Each trial consisted of first and second runs. On the first run of each trial, a mouse was forced to choose one of the arms of the T (area A1 or A2). After the mouse stayed more than 10 s, sliding doors that separated the arms (areas A1 andA2) and the connecting passage ways (areas P1 andP2) opened so that the mouse could return to the starting compartment (area S1) through the connecting passage ways by itself. The mouse was then given a 3 s delay there, followed by a free choice between the arms of the T arm. The correct answer was the other arm that had not been chosen on the first run. On day 5-8, a delay (3, 60, 120, 180 s) was applied after the first run. Data acquisition, control of sliding doors, and data analysis were performed by ImageTM software. A two-way repeated measures ANOVA was used to determine *p* value for genotype effects was determined.

### Contextual and cued fear conditioning test

A contextual and cued fear conditioning test was conducted as previously described [52, 56]. To assess fear related learning and memory, each mouse was placed in a test chamber (33 × 25 × 28 cm) with a stainless-steel grid floor (0.2 cm diameter, spaced 0.5 cm apart) (O’HARA & Co., Tokyo, Japan) illuminated at 100 ± 5 lx and allowed to explore freely for 2 min. Conditioned stimulus (55 dB white noise) was presented for 30 s, followed by a mild foot shock (2 s, 0.3 mA), which served as the unconditioned stimulus (US). Two more CS-US pairings were presented with a 2-min inter-stimulus interval. Context testing was conducted 1 day and 1 month after conditioning in the same chamber for 300 s to each mouse. Cued test with an altered context was conducted 1 day and 1 month after conditioning using a triangular box (33 × 29 × 32 cm) (O’HARA & Co., Tokyo, Japan), which was located in a different room. The test chamber was illuminated at 30 ± 5 lx. Tone stimulus for the cued test was applied for 180 s. During the test, images were captured at 1 frame per second. For each pair of successive frames, the amount of area (pixels) by which the mouse moved was measured. When this area was below a certain threshold (i.e., 30 pixels), the behavior was classified as “freezing”. The optimal threshold (amount of pixels) used to classify freezing was determined by adjusting it to the amount of freezing measured by human observation. “Freezing” that lasted less than the defined time threshold (i.e., 2 s) was not included in the analysis. Data acquisition and analysis were performed with Image FZ. Activity suppression ratio was calculated as follows: suppression ratio = (activity during testing)/(activity during baseline + activity during testing) [56]. To judge whether the differences observed were statistically significant, *p* values for genotype effects were determined either by a two-way repeated measures ANOVA (excepting for activity suppression ratio) or by a one-way ANOVA (for activity suppression ratio).

### Data analysis

All behavioral data were automatically collected using application softwares, which were derivatives of ImageJ program optimized for each type of tests by Tsuyoshi Miyakawa (available through O’HARA & Co., Tokyo, Japan). For statistical analysis, StatView (SAS Institute, Cary, NC, USA) was used. Methods for statistical analysis, including a paired *t*-test, one-way ANOVAs, and two-way repeated measures ANOVAs, are described above. Data are represented as mean ± standard error of the mean (SEM). If a *p* value was smaller than 0.05, the difference was considered statistically significant.

## Abbreviations

CC: Corpus callosum
cKO: Conditional knockout
ECM: Ectopic cortical mass
GEF: Guanine nucleotide exchange factor
GHNS: General health and neurological screens
GWAS: Genome-wide association study
KO: Knockout
RGC: Radial glial cell
